# A prognostic index for operable, node-negative breast cancer

**DOI:** 10.1038/sj.bjc.6601826

**Published:** 2004-04-27

**Authors:** M McCallum, C Baker, K Gillespie, B Cohen, H Stewart, R Leonard, D Cameron, R Leake, J Paxton, A Robertson, C Purdie, A Gould, M Steel

**Affiliations:** 1 Department of Pathology, Victoria Infirmary, South Glasgow University Hospitals NHS Trust, Glasgow, Scotland; 2 Bute Medical School, University of St Andrews, Scotland; 3 Scottish Cancer Trials Office, Edinburgh, Scotland; 4 Department of Clinical Oncology, Lothian University Hospitals NHS Trust, Edinburgh, Scotland; 5 Institute of Biomedical and Life Sciences, University of Glasgow, Scotland; 6 Department of Pathology, Ninewells Hospital and Medical School, Dundee, Scotland; 7 Cancer Intelligence Unit, Common Services Agency of the NHS (Scotland), Trinity Park House, Edinburgh, Scotland

**Keywords:** markers, immunohistochemistry, grade, BCL-2, cyclin D1

## Abstract

Clinical data and samples from patients diagnosed, more than 10 years previously, with operable node-negative breast cancer (participants in the Scottish Adjuvant Tamoxifen trial), were revisited. Cases with two distinct categories of outcome were selected; more than 10 years disease-free survival (‘good outcome’) or distant relapse within 6 years of diagnosis (‘poor outcome’). An initial set of cases was analysed for a range of putative prognostic markers and a prognostic index, distinguishing the two outcome categories, was calculated. This index was then validated by testing its predictive power on a second, independent set of cases. A combination of histological grade plus immunochemical staining for BCL-2, p27 and Cyclin D1, generated a useful prognostic index for tamoxifen-treated patients but not for those treated by surgery alone. The value of the index was confirmed in a second set of tamoxifen-treated, early stage breast cancers. Overall, it correctly predicted good and poor outcome in 79 and 74% of cases, respectively (odds ratio 11.0). Other markers assessed added little to prediction of outcome. In the case of molecular assays, sensitivity and reliability were compromised by the age of the tissue specimens and the variability of fixation protocols. In selecting patients for adjuvant systemic chemotherapy, the proposed index improves considerably on current international guidelines and matches the performance reported for ‘gene-expression signature’ analysis.

There is long-standing controversy over the management of small, node-negative breast cancers. Surgical excision, with or without local radiotherapy, plus adjuvant tamoxifen will result in long-term disease-free survival for some 80% of patients. On the other hand, around 20% will develop distant metastases and this figure can be reduced by adjuvant chemotherapy (Early Breast Trialists, 1998). The issue is whether it is justifiable to expose all patients with early disease to the side effects of cytotoxic therapy for the benefit of the minority. If those at greatest risk of relapse could be identified at the time of diagnosis, treatment decisions would be simplified. Many attempts have been made to categorise patients with operable node-negative tumours according to their individual prognoses (Merkl and Osborne, 1989; Leonard, 1999; Isaacs *et al*, 2001; Mirza *et al*, 2002; Niu *et al*, 2002) but no single marker or combination of markers has gained widespread acceptance and authoritative guidelines achieve only very broad selection of ‘higher risk’ patients for adjuvant cytotoxic chemotherapy (Eifel *et al*, 2001; Goldhirsch *et al*, 2001). Most recently, a prognostic ‘gene-expression signature’ has been described, based on microarray analysis of 70 genes (van't Veer *et al*, 2002; van de Vijver *et al*, 2002). However, it will be some time before such sophisticated technology can be applied in routine diagnostic practice (Schubert, 2003). Meanwhile, the range of possible predictive indicators, identifiable by long-established techniques, continues to grow and the potential benefits of a simple and reliable prognostic index justify further assessment.

The natural history of breast cancer means that rigorous evaluation of prognostic markers for ‘early’ disease requires complete follow-up of substantial numbers of fully documented patients over very long periods. The rarity of such databases is a recognised limiting factor. However, the Scottish Adjuvant Tamoxifen trial, which recruited patients with good prognosis between 1980 and 1984, meets many of the necessary criteria (Breast Cancer Trials Committee, 1987; Stewart *et al*, 2001). In total, 751 of the patients studied had ‘operable’ disease, defined as small (typically <2 cm) mobile primary tumours with no pathological involvement of axillary lymph nodes. All were treated by mastectomy before randomisation to adjuvant tamoxifen for 5 years or to observation, with tamoxifen on first relapse. Clinical and pathological findings were recorded in a standardised fashion and follow-up has been meticulous, with outcome data available on over 98% of the original entrants (Stewart *et al*, 2001). Of these 751 patients, 16% considered potentially curable by surgery alone, had suffered distant relapse within 6 years of diagnosis.

The aim of the present study was to compare cases of operable node-negative breast cancer, matched by time and place of diagnosis and treatment, but differing by outcome, for a range of putative prognostic markers and to derive empirically an index that distinguished most accurately patients destined for long-term disease-free survival from those who would suffer early distant relapse. That index would then be tested ‘prospectively’ on a second, independent set of tumours for which the same outcome data were available.

The tumour characteristics assessed included prognostic indicators such as histopathological grade, tumour type and oestrogen receptor alpha (ER), which are almost universally applied, plus the following less well-established markers, chosen on the basis of published reports.

## 

### By immunohistochemistry

#### Progesterone receptor (PgR)

This is usually dependent on a functioning oestrogen receptor, though there are exceptions. The combination of ER and PgR may therefore provide better prognostic discrimination than either on its own (Yoo *et al*, 1997; Yasui and Potter, 1999).

#### Cyclin D1

Although amplification of a region of chromosome 11 (q13) is a common finding in breast cancer and the most consistently overexpressed oncogene from this region is Cyclin D1, there is evidence that Cyclin D1 overexpression is a favourable prognostic sign, associated with ER-positive, well-differentiated tumours (Barnes and Gillett, 1998).

#### Ki67

The antigen expressed by this antibody is closely associated with cell proliferation. Levels of Ki67 positivity have been correlated with other features of aggressive tumour growth and hence with poor prognosis (Forrest, 1997).

#### P27

This gene product is an inhibitor of cyclin-dependent kinases and hence functions as a tumour suppressor. Barnes and Gillett (1998) found some association between levels of Cyclin D1 and p27, both correlating with favourable outcome. Porter *et al* (1997) confirmed that p27 expression correlated positively with survival in young breast cancer patients.

#### BCL-2

The protein product of BCL-2 is located primarily within the mitochondrial membrane and appears to function as an inhibitor of cytochrome-*C* release, thus preventing initiation of one apoptotic pathway. It is classified as an oncogene and was initially identified as an important causal factor in certain B-cell lymphomas. However, its role in breast cancer seems to be more complex. While breast tumours expressing high levels of BCL-2 are reported to show low rates of apoptotic cell death, they also have low proliferation rates, low histopathological grades, absence of p53 mutations and improved survival (Charpin *et al*, 1998; van Slooten *et al*, 1998; Le *et al*, 1999).

#### Urokinase plasminogen activator (UPA)

Digestion of the surrounding tumour matrix by endogenous proteases may be an important mechanism whereby carcinomas invade and metastasise. UPA is one of the proteases linked to poor outcome in breast cancer (Dano *et al*, 1985; Duffy *et al*, 1990; Bouchet *et al*, 1998).

### By nuclear DNA content

Both aneuploidy and a high S-phase fraction have been recorded as indicators of poor prognosis in early breast cancer, although the latter may simply replicate the information available from histological measurement of mitotic index or from immunohistochemical markers of proliferation (Merkel and Osborne, 1989; Bagwell *et al*, 2001).

### By molecular analysis

Allele imbalance at specific loci, implying amplification or loss of one copy of a particular DNA sequence has been correlated with prognosis in a number of studies. Among the loci most consistently implicated are 11q13, 13q12–13 and 17p13 (van de Vijver, 1993; Devillee and Cornelisse, 1994).

## MATERIALS AND METHODS

### Case selection

Records of the Scottish Adjuvant Tamoxifen Trial were accessed through the Cancer Intelligence Unit of the NHS (Scotland) Common Services Agency in Edinburgh. For the initial (‘index-generating’) data set, patients from both the Tamoxifen and the control arms of the trial were included. Cases were classified as ‘good’ outcome if the patient had survived, without evidence of disease, for at least 10 years and as ‘poor’ outcome if distant metastases had been recorded within 6 years of diagnosis. To enhance the power of the study, case selection was deliberately biased towards the poor outcome group, who would otherwise have formed less than 20% of the total series. The second (‘validation test’) set of samples, comprised only patients who had received adjuvant tamoxifen and, to avoid a shortfall in ‘poor’ outcome cases, those drawn from the Tamoxifen Trial series were supplemented from two Scottish breast cancer centres (Victoria Infirmary, Glasgow and Ninewells Hospital, Dundee) where data had been recorded in uniform manner for over 10 years. ‘Good’ and ‘poor’ outcomes were defined as for the first series. There were thus six subsets of patients, four in the initial series and two in the second ‘test’ series (Table 1

**Table 1:** Breakdown of patient groups

		**Adjuvant tamoxifen**	**No adjuvant tamoxifen**
	Good		
	outcome	23	25
First			
set			
	Poor		
	outcome	21	14
			
	Good		
	outcome	41	
Second			
(test) set			
	Poor		
	outcome	24	
Subtotals	Good outcome	64	25
	Poor outcome	45	14
			
Total		109	39

). Overall, 89 had remained disease-free for more than 10 years, while 59 (40%) had developed distant metastatic disease within 6 years of diagnosis.

### Tissue samples and histology

Original tumour and lymph node blocks were located and retrieved from hospital pathology departments. In a number of instances, insufficient tumour material remained for the studies proposed and, where possible, another case, from the same trial arm and with the same outcome, was substituted. Fresh sections were cut, mounted, stained with haematoxylin and eosin and re-examined by a single specialist breast pathologist, who assessed tumour type, histological grade (nuclear pleomorphism, mitotic index and tubule formation), according to standard criteria (Bloom and Richardson, 1957; Elston and Ellis, 1991). Degree of necrosis, vascular invasion and extent of lymphocyte infiltration were also evaluated. Where different regions of the specimen revealed tumour of differing grades, the highest grade was recorded.

### Immunohistochemistry

The primary antibodies used to demonstrate each of the listed markers are shown in Table 2

**Table 2:** Primary antibodies used in immunohistochemical analyses

**Specificity**	**Antibody**
Oestrogen receptor	Dako ID5 M7074
Progesterone receptor	Novocastra NCL-PGR
Cyclin D1	Santa Cruz SC 6281
P27	Santa Cruz SC 1641
BCL-2	Dako M0887
Ki67	Dako A0047 (Rabbit polyclonal)
Urokinase	From Dept of Chemical Pathology
plasminogen activator	Academic Hospital, Nijmegen
	(Rabbit polycloonal)

. All, except anti-Ki67 and anti-UPA, are mouse monoclonal products.

Protocols were optimised for each antibody but, in general, 5 *μ*m sections of each tumour were mounted onto APES-coated slides, dewaxed in Histoclear™, immersed in 3% hydrogen peroxide in methanol for 15 min (to inhibit endogenous peroxide activity) then exposed to ‘antigen-retrieval’ processing (2 × 10 min in a 750 W microwave in citrate buffer pH 6). Nonspecific antibody binding was blocked by 20 min immersion in normal goat serum (20% in PBS). Between treatments slides were rinsed in water.

The relevant monoclonal or polyclonal primary antibody was applied at the predetermined optimal concentration overnight at room temperature. After rinsing, biotinylated F(ab)′_2_ fragment of goat anti-mouse Ig (goat anti-rabbit for polyclonal primary antibodies) diluted 1 : 200 in PBS at pH 7.6 was applied for 30 min at room temperature, followed by peroxidase-conjugated streptavidin, diluted 1 : 300 in PBS, for 30 min at room temperature. After a further rinse in PBS, peroxidase activity was detected by incubating in freshly prepared Diaminobenzidine solution for 10 min. Further rinses were followed by counterstaining with haematoxylin. Sections were finally dehydrated, dried and mounted for microscopic examination.

Results were recorded as the percentage of invasive cancer cells showing specific staining. In general, all degrees of staining were recorded but, where only overexpression was considered relevant, positive scoring was restricted to those tumour cells showing staining intensity above that of the adjacent benign epithelial cells.

To minimise subjectivity in this assessment, for BCL-2 assays, two histopathologists scored each slide until training established a high level of concordance between them. For p27 and Ki67, scoring was undertaken by a single observer, repeating the analysis without reference to the first result. Where there was greater that 10% discrepancy between the two results, reading was repeated until a consistent score was obtained. For Ki67, the range of % positive cells was low and a more accurate assessment was considered necessary. A graticule was therefore used to identify random high power (× 400) fields, within which counting continued until a total of at least 200 tumour cells was achieved. A positive control was included in each batch of staining, being a section from a breast cancer known to stain strongly with the given antibody. A negative control was stained along with each test section and consisted of the adjacent section treated identically except that no primary antibody was applied.

### DNA content

Following published techniques (Hedley, 1989; Bagwell *et al*, 2001), intact nuclei were extracted from 20 *μ*m sections of tumour after trimming to remove adjacent nontumour tissue. Thick sections of normal lymph node were used as sources of diploid nuclei for reference. The DNA was stained with propidium iodide and content recorded by flow cytometry on at least 10 000 nuclei per sample. The resulting histogram was analysed by a standard FACScan program, which determined the percentage of diploid cells, the percentage in S phase (‘S-phase fraction’) and the overall DNA index (diploid, hypo- or hyperploid).

### Molecular analysis

PCR amplification of microsatellites D11S534, D11S970, D13S267, D13S171 and D17S1322 was undertaken on DNA extracted from thin sections of fixed tissue (both tumour and normal lymph node from the same patient), using a commercial extraction kit (Nuclear Biosciences). PCR products were denatured and separated on an 8% polyacrylamide sequencing gel, transferred to a nylon membrane and probed with a ^32^P-labelled (CA)_22_ oligonucleotide. Signals were visualised by autoradiography and the relative abundance of each allele in tumour and normal tissue compared by eye.

### Statistical methods

The associations of the individual histological, immunohistochemical and molecular markers with outcome were examined by comparing their values between the two outcome groups for the first set of patients. Patients from the adjuvant tamoxifen and the control arms of the trial were analysed separately.

For intrinsically categorical variables (tumour type, histological grade) the *χ*^2^-test was used to assess associations with outcome, and the categories grouped to identify the best categorisation for a prognostic index.

For continuous variables (age, degree of necrosis, vascular invasion, extent of lymphocyte infiltration, DNA content, ER, PgR, UPA, Ki67, BCL-2, Cyclin-D1, p27), Student's *t*-test was used in the first instance to assess association. For those variables showing a significant association with outcome, a range of thresholds for categorising the patients was tried and the *χ*^2^ test used to choose the best categorisation for prognosis.

The variables most strongly associated with outcome were combined empirically to form a prognostic score. For each patient the score was calculated and a threshold for good *vs* poor prognosis was derived for each of the trial arms.

The scoring system was validated by applying it to the second set of patients. The proportions of good and poor prognosis patients correctly classified were calculated. The odds ratio for the score between the good and poor outcome cases was calculated for the adjuvant tamoxifen patients from first and second sets combined.

Decision tree analysis (Venables and Ripley, 2002) was finally applied to the whole data set as an additional independent test of the findings. This stepwise approach splits the set of patients into groups by every possible threshold value of every putative prognostic factor, and chooses the split which results in the maximum number of patients correctly classified. Next, the ‘best’ split is chosen for each of the two resulting subgroups. The process is repeated until no further split can be found that results in subgroups with different outcome. To avoid overfitting of the model, which would generate an overoptimistic result, cross-validation, by repeated holding-out of 10% of the patients, was incorporated in the analysis. S-Plus statistical software was used.

Among the advantages of decision tree analysis over more conventional approaches, such as logistic regression, are that (a) it deals easily with interactions among the prognostic variables and with nonmonotonic relationships between the predictors and the outcome, (b) subjects with missing data can be classified using the data that are available for them, and (c) the results are intuitively easy to interpret.

## RESULTS

It was recognised that molecular analysis of samples fixed up to 20 years earlier and using various modifications of a standard formalin-based protocol, would present difficulties. In the event, analysable microsatellite products were generated from 58 to 74% of samples, depending on the primers used. Heterozygosity rates varied from 50 to 73% and allele imbalance in tumour tissue was recorded in 23–37.5% of informative cases. Furthermore, for individual informative tumours, there was measurable concordance for allele imbalance between contiguous loci but not between loci on different chromosome arms. These encouraging results were, however, offset by the finding of what appeared to be ‘new’ alleles in a number of tumours. Since, in our experience, and that of others, microsatellite instability is rare in breast cancers, we suspected that the multiple (35) rounds of PCR amplification required to obtain sufficient product from fixed tissue sections were generating artefacts. This was confirmed in a formal comparison of fixed and fresh tissue from the same source (Cohen *et al*, 1998) and the problem was not resolved by varying technical conditions or the type of polymerase used. Regretfully, therefore, the molecular findings could not be used to derive a prognostic index.

The ‘good outcome’ group were younger (mean age 53.0 years, 95% CI 50.01–55.99 *vs* mean age 57.7 years, 95% CI 54.24–61.25. *P*=0.044). The requirement for 10 years survival, in order to be included in the ‘good’ prognosis category, probably contributed to this effect.

Of the 83 tumours in the initial set, 13 were of ‘special’ pathological types (tubular or lobular) and there was no difference in their distribution among subgroups. The presence of ductal (or, in one instance, lobular) carcinoma *in situ* was correlated weakly with good outcome (*P*=0.03) and, while this association failed to reach statistical significance for either arm of the trial, on its own, it was stronger for the subgroup which had not received adjuvant tamoxifen. As anticipated, ‘good’ and ‘poor’ outcome cases differed on overall pathological grade, with 30% of the former, but 54% of the latter, being grade 3 (*P*=0.025). Unexpectedly, this difference was accounted for entirely by cases from the adjuvant tamoxifen arm of the trial, where the proportions of grade 3 tumours in the good and poor outcome group were 26 and 71%, respectively (*P*=0.003). No significant differences were found in extent of vascular invasion, nor of tumour necrosis. Lymphocytic infiltration was sparse or absent in the majority of cases. It was substantial in only four tumours, all of which belonged to the ‘good outcome, no adjuvant tamoxifen’ category. However, numbers were too small to justify incorporating this criterion into a general prognostic index.

On DNA content analysis, good outcome tumours were more often entirely diploid (46 *vs* 36.5%) but the difference was not significant. Similarly, the distribution of hyper and hypodiploid tumours showed no association with outcome. For cases showing any degree of aneuploidy, there was a trend towards a higher proportion of aneuploid cells among good prognosis tumours but, again, this did not achieve significance. S-phase fractions, both for the aneuploid and the diploid component of given tumours, varied widely (2–50%) but neither correlated with prognostic group.

Among the immunohistochemical markers, oestrogen receptor was more likely to be completely negative in the poor outcome tumours (55 *vs* 41%) but the difference was nonsignificant. Predictably, the difference was more marked for the groups who had received adjuvant tamoxifen, where 44% of the good outcome, but 67% of the poor outcome cases, had no detectable oestrogen receptor (*P*=0.04). Interestingly, the immunohistochemical results correlated poorly with the original records of ER level, which had been determined by a dextran-coated charcoal exchange assay (Breast Cancer Trials Committee, 1987), probably because the earlier technique was highly sensitive to delay in sample processing (R Leake, personal communication). Progesterone receptor distribution was indistinguishable across the subgroups. The same was true for UPA and Ki67 scores. Furthermore, 20% of the Ki67 slides gave results considered to be technically unsatisfactory, a problem that was not encountered with any of the other antibodies.

Positivity for BCL-2 was clearly associated with a good outcome, whether treating percentage of positive cells as a continuous variable (*P*=0.009) or taking a value of >5% of cells stained as the boundary for a *χ*^2^-test (*P*=0.01). The same trend was apparent for Cyclin D1 and p27 staining but in neither case was there a statistically significant result (*P*-values 0.06–0.15). For p27, the association was restricted to the subgroup that had received adjuvant tamoxifen and for Cyclin D1, the correlation was stronger for the same subgroup.

Thus, of all the putative markers assessed, those that appeared, individually, to merit further scrutiny, with a view to constructing a prognostic index, were:

- Tumour grade.
- Presence of carcinoma *in situ*.
- Immunohistochemical staining for:
  - BCL-2
  - ER
  - p27
  - Cyclin D1.

Only BCL-2 staining and carcinoma *in situ* seemed promising for patients who had not received adjuvant tamoxifen. In that subgroup, the combination of low/negative BCL-2 (<30% cells staining) and absence of carcinoma *in situ* was seen in 50% of those with poor, but only 12.5% of those with good outcome. Overall accuracy was 74% (odds ratio 7.0, *P*=0.011). In terms of outcome prediction, the combination was superior to either assay on its own (odds ratios 2.95 and 3.5; *P*-values 0.13 and 0.08).

Given that each putative marker was being assessed for its correlation with outcome in the same set of tumours, those showing any prognostic potential were bound to correlate, to some extent, with each other. It has already been shown, for example, that overexpression of BCL-2 is strongly dependent on a functioning ER (Le *et al*, 1999; Burow *et al*, 2000). Therefore, to minimise the total number of variables comprising a prognostic index for the subgroup who had received adjuvant tamoxifen, a series of pairwise comparisons was undertaken to determine the independent predictive value of each of the above six markers. This demonstrated that pathological grade and BCL-2 staining were the most powerful independent contributors to any combination, while carcinoma *in situ* and oestrogen receptor status added least information. Predictions based on tumour grade, or immunohistochemical staining for BCL-2, p27 or cyclin D1, all showed a useful measure of independence from each other. A prognostic index was then derived by classifying the results obtained for each of the four markers into three categories. These are already specified for pathological tumour grade and, on viewing the raw data for the others, the same cutoff values could be assigned to them all, such that ⩾70% positive staining = ‘strong’, 31–69%= ‘moderate’ and ⩽30% = ‘weak’ (see Table 3

**Table 3:** Distribution of prognostic markers

		**Grade**			**er %**		**bcl2 %**			**Cyclin D1 %**			**p27 %**		
		**1**	**2**	**3**	**>20**	**<20**	**>70**	**31–69**	**<30**	**>70**	**31–69**	**<30**	**>70**	**31–69**	**<30**
Tumour set 1 (On Tam)	GOOD	11	6	6	11	11	7	4	12	8	6	9	8	3	10
	POOR	5	1	15	6	13	3	1	16	5	2	13	2	3	12
Tumour set 1 (Not on Tam)	GOOD	9	7	8	13	9	10	3	12	9	5	9	4	6	9
	POOR	6	4	4	8	5	3	1	10	6	1	7	4	5	3
Tumour set 2 (On Tam)	GOOD	20	11	9	31	10	15	5	21	33	1	7	21	5	15
	POOR	1	11	12	12	12	1	5	18	9	1	14	6	4	14
TOTAL (On Tam only)	GOOD	31	17	15	42	21	22	9	33	41	7	16	29	8	25
	POOR	6	12	27	18	25	4	6	34	14	3	27	8	7	26

). Following the convention for pathological grading, a ‘strong’ result was assigned a numerical score of 1, a ‘moderate’ result, 2 and a ‘weak’ result, 3 (Figure 1

**Figure 1:**
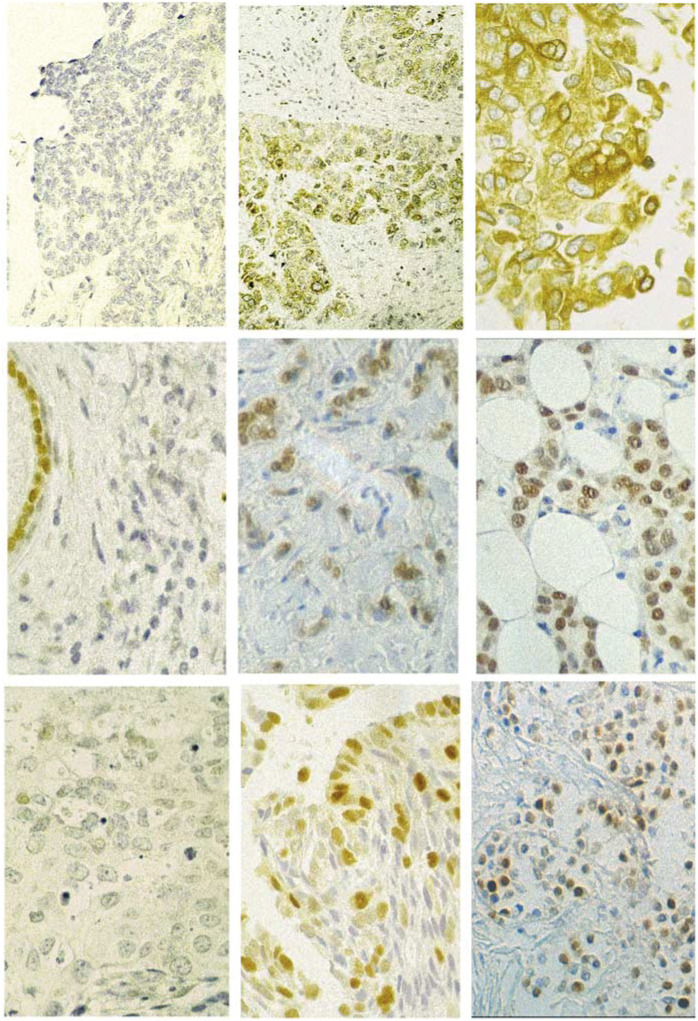
Photomicrographs illustrating ‘weak/negative’. Moderate and ‘strong’ staining for BCL-2, Cyclin D1 and p27. Note the positive staining of normal duct epithelium in the Cyclin D1 ‘negative’ section.

). Adding these scores for all four markers produced an aggregate score of between 4 and 12 for any given tumour.

The findings are summarised in Tables 3 and 4

**Table 4:** Performance of prognostic index in patients receiving adjuvant tamoxifen

		
**(a) First set**	**Good outcome**	**Poor outcome**
Aggregate		
Score <10	17	5
Aggregate		
Score ⩾10	6	14
		
		
**(b) Second set**	**Good outcome**	**Poor outcome**
Aggregate		
Score <10	36	9
Aggregate		
Score ⩾10	4	15
		
		
**(c) Combined sets**	**Good outcome**	**Poor outcome**
Aggregate		
Score <10	53	14
Aggregate		
Score ⩾10	10	29

Aggregate scores are derived from Path grade, plus extent of staining for BCL-2, Cyclin D1 and p27, with individual scores of 1, 2 or 3 for each marker, as described in the text.

. As shown in Table 4, an aggregate score ⩾10 provided efficient discrimination between poor and good outcome patients, correctly identifying 31 of 42 tumours (74%), for which all data were available (odds ratio 7.93, 95% CI 1.99, 31.59, *P*=0.0021). For three patients (two good outcome, one poor) a single value was missing from the set but they were included in the analysis because substituting any score (1, 2 or 3) for the missing one would not have brought the aggregate to more than nine or less than 10, respectively. Varying the weighting of the different parameters (e.g. increasing the contribution of path grade and BCL-2, the two strongest individual predictors of outcome) did not improve overall performance of the index, nor did varying the cutoff values used to define ‘strong’, ‘moderate’ and ‘weak’ categories for any of the markers, nor did adding further data, such as ER score or presence of carcinoma *in situ*.

The test of any index derived from retrospective data must be to apply it prospectively. In formal terms, we were able to do this by ‘blinding’ a second series of tumours. Because tamoxifen was used very widely for early breast cancer in Scotland from the mid-1980s, it was not possible to assemble sufficient unexposed, poor outcome, cases to validate the index based on BCL-2 positivity and presence of carcinoma *in situ*. However, 65 samples were collected from patients with operable node-negative breast cancers, diagnosed before 1990, who had received adjuvant tamoxifen and had been followed up for at least 10 years or until death. Overall, 41 had remained free from disease, while 24 had suffered distant relapse within 6 years of diagnosis.

Fresh sections were cut and reassessed by the same specialist breast pathologist. Immunohistochemical assays were carried out and scored as before. On applying the prognostic index, derived as above for the adjuvant tamoxifen group, it correctly identified 36 of the 40 (90%) ‘good’ outcome and 15 of the 24 (62.5%) ‘poor’ outcome tumours for which complete data were obtained, an overall accuracy rate of 79.7% (odds ratio 15.0, 95% CI 4.0, 56.3, *P*=<0.0001) (Table 4).

Differences in performance of the index between the initial and the ‘validation’ series of cases were not significant. Combining the two data sets (Table 4c), 29 of 43 tumours (67.4%) that relapsed early, and 53 of 63 (84.1%) that had not recurred after 10 years, were correctly identified (overall accuracy 77.4%; odds ratio 11.0, 95% CI 4.3, 27.8, *P*<0.0001). The best of the individual predictive markers (path grade 3 *vs* <3) had an overall accuracy of 69% (odds ratio 4.7; *P*=0.0002).

Cross-validated decision tree analysis was applied separately to the 109 adjuvant tamoxifen patients and the 39 from the control arm of the trial.

**Figure 2:**
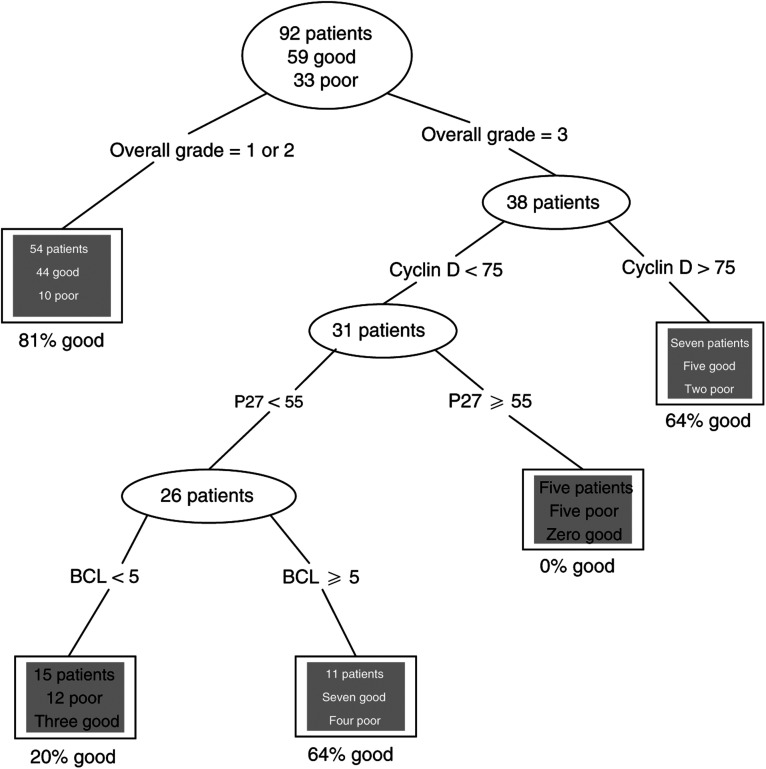
Classification tree for tamoxifen-treated patients.

For the tamoxifen-treated group, the resulting decision tree is shown as Figure 2

. As 17 of the 109 patients had incomplete data, only 92 patients were used to ‘grow’ the tree. The variables included in this classification process are overall tumour grade, Cyclin D1, p27 and BCL-2. This tree correctly classifies 79% of all the patients; 95% of those with good outcome and 56% of those with poor outcome (see Table 5

**Table 5:** Decision tree performance

		**Actual outcome**	
		**Good**	**Poor**
Predicted by	Good	61	**20**
Decision tree	Poor	**3**	25
	Total	64	45

Odds ratio 25, 95% confidence interval (7, 93). This table refers to the decision tree for tamoxifen-treated patients, shown in Figure 1. It incorporates the 17 patients excluded from the tree growing because of missing data. Their predictions are based on the data they do have. Misclassified cases are shown in bold.

).

For patients not receiving tamoxifen, numbers were smaller, which affected the reliability of splits. Age was a useful predictor (those over 45 had better prognosis), as were presence of carcinoma *in situ* and BCL-2 score.

## DISCUSSION

Features of the present study include substantial cohort size of node-negative patients (particularly of those with poor outcome), long and complete follow-up, consistent clinical management and validation of initial findings, by ‘prospective’ application and by independent ‘decision tree’ analysis. The latter statistical procedure identifies essentially the same parameters contributing to prediction of outcome and, although the information is handled differently, the end results, in terms of prognostic accuracy, are similar. The potential impact of the prognostic index on clinical practice can be calculated as follows.

If 1000 patients with stage one breast cancer were treated by surgery, local radiotherapy and tamoxifen alone, around 200 would suffer distant relapse. These 200 should therefore be candidates for systemic adjuvant chemotherapy. The proposed index would identify 135 of them (67.4%), but would (wrongly) identify a further 127 ‘cured’ patients as at high risk of relapse (15.9% of 800). On this basis, 262 (26.2%) of the 1000 patients would receive cytotoxic drugs, just over half of them appropriately. Overall, 12.7% of the patients would be exposed needlessly to systemic cytotoxic agents and 6.5% would ‘miss out’ on potentially beneficial therapy. Applying the same analysis to predictions based on gene expression signature, and considering only node-negative patients (van de Vijver *et al*, 2002), 91.3% of those with poor outcome would be correctly identified, but at the expense of including 41.4% of those with good outcome. Thus, 514 of the 1000 cases would have received adjuvant chemotherapy. Of the 200 destined for early relapse, 183 would have been included. Reducing the cutoff aggregate value for our prognostic index from 10 to 8 has the effect of identifying 86% of those with poor, but including 44% of those with good outcome. On that basis, we would give adjuvant treatment to 524 of our notional 1000 patients and would ‘miss’ only 28 who actually require it.

None of these scenarios represents a perfect situation, but each is, arguably, an advance on current NCI or St Gallen guidelines (Eifel *et al*, 2001; Goldhirsch *et al*, 2001), both of which recommend that adjuvant chemotherapy be given to the great majority of ‘early’ breast cancer patients. Can our prognostic index (or others) be improved? The selection of individual prognostic markers is debatable. We chose not to include, for example, p53 or HER2, both of which have been studied extensively, but which have not been found consistently useful as independent predictors of outcome in early breast cancer (Pharoah *et al*, 1999; Mirza *et al*, 2002; Schlotter *et al*, 2003), although they may be more accurate for node-positive cases (Sjogren *et al*, 1998; Pharoah *et al*, 1999) Cyclin E, currently exciting much interest (Keyomarsi *et al*, 2002; Loden *et al*, 2002), was, perhaps incorrectly, excluded on the grounds that its expression was related to that of p27 (Porter *et al*, 1997). E-cadherin, *c*-myc and p21 are other markers not included in this study that might be assessed as potential components of a more refined prognostic index (Heimann *et al*, 2000; Schlotter *et al*, 2003; Winters *et al*, 2003). Several markers reported by others to be of predictive value were not so in our hands. The fact that we were dealing with tissues fixed in formalin (according to a variety of protocols) up to 20 years earlier, accounts for the unreliability of allele imbalance studies and may also have affected flow cytometry analyses. Most positive results reported to date for UPA have relied upon tumour cytosol protein extracts (Bouchet *et al*, 1998) so that the immunohistochemical assay we applied is relatively untried. It is not clear why Ki67 gave such disappointing results; however, a recent review of prognostic factors in node-negative breast cancer notes that ‘lack of standardisation in measurement techniques for many of the markers, including Cathepsin D, Ki67, Her2/neu and p53 limited their current usefulness’ (Mirza *et al*, 2002).

Any prognostic system must be tested and retested in different centres, by different operators and on different patient cohorts before it can be applied with confidence to clinical management. In the course of such development, there is scope for addition, or substitution of new markers. Those markers that did generate predictive information from our archival specimens are, *ipso facto*, likely to prove robust components of a prognostic panel for wider application but others may prove superior.

It is almost inevitable that indicators of prognosis will be influenced by treatment (Borg *et al*, 2003) since responses to both hormonal and chemotherapy are affected by the biological characteristics of a given tumour. Our finding that different indices apply to patients in the tamoxifen and the control arms of the trial is therefore no surprise and, in the strictest sense, our index is predictive (of response to tamoxifen) rather than truly prognostic. However, tamoxifen is now so widely used, and so well tolerated, in early breast cancer that applicability of the index as a prognostic tool is not seriously compromised.

The issue of predictive markers (i.e. identifying how individual patients will respond to specific anticancer therapies) is important but separate. For ‘early stage’ cancers it should arise only after identification of the subset that will not do well on surgery and tamoxifen alone. For those with nodal or distant spread, it is an immediate concern on diagnosis. The recognition that breast cancers are heterogeneous in terms of sensitivity to radiation and to different drugs or drug combinations – and hence the ability to ‘tailor’ treatment to the individual patient – gives promise of major therapeutic advances. Several molecular and immunohistochemical predictive protocols are now being evaluated (Ross and Fletcher, 1999; Lonning *et al*, 2001; Bertucci *et al*, 2002; Sotirou *et al*, 2002; Chang *et al*, 2003; Egawa *et al*, 2003; Moliterni *et al*, 2003; Winters *et al*, 2003; Robson *et al*, 2004).

Gene expression microarray technology is also being applied to prognosis in the same setting as the present study. It would therefore be of great interest to compare ‘gene-expression signature’ (van de Vijver *et al*, 2002) with our index in comparable patient groups. The two approaches appear to provide comparable levels of sensitivity and specificity. Gene expression profiling has the potential to provide rapid, comprehensive and relatively objective information, but requires fresh tumour tissue and ‘high tec’ facilities, currently available in only one or two specialist centres (Schubert, 2003). While the approach we have illustrated here may be time-consuming and require experience to minimise subjectivity, it can be applied in any diagnostic histology laboratory.

## References

[bib1] Bagwell CB, Clark GM, Spyratos F, Chassevent A, Bendahl PO, Stal O, Killander D, Jourdan ML, Romain S, Hunsberger B, Wright S, Baldetorp B (2001) DNA and cell cycle analysis as prognostic indicators in breast tumours revisited. Clin Lab Med 21: 875–895 11770293

[bib2] Barnes DM, Gillett CE (1998) Cyclin D1 in breast cancer. Breast Cancer Res Treat 52: 1–15 10066068 10.1023/a:1006103831990

[bib3] Bertucci F, Houlgatte R, Granjeaud S, Nasser V, Lorioid B, Beaudoing E, Hingcamp P, Jacquemeier J, Viens P, Birnbaum D, Nguyen C (2002) Prognosis of breast cancer and gene expression profiling using DNA arrays. Ann NY Acad Sci 975: 217–231 12538167 10.1111/j.1749-6632.2002.tb05954.x

[bib4] Bloom HJB, Richardson WW (1957) Histological grading prognosis in breast cancer: study of 409 cases of which 359 have been followed up for 15 years. Br J Cancer 11: 35–46 10.1038/bjc.1957.43 PMC2073885 13499785

[bib5] Borg A, Ferno M, Peterson C (2003) Predicting the future of breast cancer. Nat Med 9: 16–18 12514712 10.1038/nm0103-16

[bib6] Bouchet C, Spyratos F, Hacene K, Durcos L, Becette V, Oglobine J (1998) Prognostic value of urokinase plasminogen activator in primary breast carcinoma: comparison of two immunoassay methods. Br J Cancer 77: 1495–1501 9652768 10.1038/bjc.1998.246 PMC2150205

[bib7] Breast Cancer Trials Committee, The Scottish Cancer trials Office (1987) Adjuvant tamoxifen in the management of operable breast cancer: the Scottish trial. Lancet ii: 171–175 2885637

[bib8] Burow ME, Weldon CB, Tang Y, McLachlan JA, Beckman BS (2000) Oestrogen-mediated suppression of tumour necrosis factor alpha-induced apoptosis in MCF-7 cells: subversion of BCL-2 by anti-estrogens. J Steroid Biochem Mol Biol 78: 409–418 10.1016/s0960-0760(01)00117-0 11738551

[bib9] Chang JC, Wooten EL, Tsimelzon A, Hilsenbeck SG, Gutierrez MC, Elledge R, Mohsin S, Osborne CK, Chammers GC, Allred DC, O'Connell P (2003) Gene expression profiling for the prediction of therapeutic response to docitaxel in patients with breast cancer. Lancet 362: 362–369 12907009 10.1016/S0140-6736(03)14023-8

[bib10] Charpin C, Garcia S, Bouvier P, Devictor B, Andrac L, Lavaut MN, Allasia C (1998) BCL-2 automated and quantitative immunocytochemical assays in breast carcinomas: correlation with ten year follow-up. J Clin Oncol 16: 2025–2031 9626199 10.1200/JCO.1998.16.6.2025

[bib11] Cohen BB, Anderson VA, Gillespie C (1998) Artefactual allotyping related to DNA source, concentration and the number of PCR cycles. Dis Markers 14: 165–167 10427476 10.1155/1998/629481 PMC3851338

[bib12] Dano K, Andreasen PA, Grondahl-Hansen J, Kristensen P, Nielsen LS, Skriver L (1985) Plasminogen activators, tissue degradation and cancer. Adv Cancer Res 44: 139–266 2930999 10.1016/s0065-230x(08)60028-7

[bib13] Devillee P, Cornelisse CJ (1994) Review: somatic genetic changes in human breast cancer. Biochim Biophys Acta 1198: 113–130 7819270 10.1016/0304-419x(94)90009-4

[bib14] Duffy MJ, Reilly D, O'Sullivan C, O'Higgins N, Fennelly JJ, Andreasen P (1990) Urokinase plasminogen activator, a new and independent prognostic marker in breast cancer. Cancer Res 50: 6827–6829 2119883

[bib15] Early Breast Cancer Trialists' Collaborative Group (1998) Polychemotherapy for early breast cancer: an overview of the randomised trials. Lancet 352: 930–942 9752815

[bib16] Egawa C, Motimura K, Miyoshi Y, Takamura Y, Taguchi T, Tamaki Y, Inaji H, Koyama H, Noguchi S (2003) Increased expression of BRCA1 mRNA predicts favorable response to anthracycline-containing chemotherapy in breast cancers. Breast Cancer Res Treat 78: 45–60 12611456 10.1023/a:1022101310500

[bib17] Eifel P, Axelson JA, Costa J, Crowley J, Curran Jr WJ, Deshler A, Fulton S, Hendricks CB, Kemeny M, Kornblith AB, Louis TA, Markman M, Mayer R, Roter D (2001) National Institutes of Health Consensus Development Conference Statement: adjuvant therapy for breast cancer, November 1–3, 2000. J Natl Cancer Inst 93: 979–989 11438563 10.1093/jnci/93.13.979

[bib18] Elston CW, Ellis IO (1991) Pathological prognostic factors in breast cancer. I. The value of histological grade in breast cancer: experience from a large study with long-term follow-up. Histopathology 19: 403–410 1757079 10.1111/j.1365-2559.1991.tb00229.x

[bib19] Forrest APM (1997) Introduction to breast cancer. In: Biology of Female Cancers, SP Langdon,WR Miller and A Berchuck (eds), pp 31–42. Boca Raton, FL: CRC Press

[bib20] Goldhirsch A, Glick JH, Gelber RD, Coates AS, Senn HJ (2001) Meeting highlights: International Consensus Panel on the Treatment of Primary Breast Cancer: Seventh International Conference on Adjuvant Therapy of Primary Breast Cancer. J Clin Oncol 19: 3817–3827 11559719 10.1200/JCO.2001.19.18.3817

[bib21] Hedley DW (1989) Flow cytometry using paraffin-embedded tissue: five years on. Cytometry 10: 229–241 2653738 10.1002/cyto.990100302

[bib22] Heimann R, Lan F, McBride R, Hellman S (2000) Separating favorable from unfavorable prognostic markers in breast cancer: the role of E-cadherin. Cancer Res 60: 298–304 10667580

[bib23] Isaacs C, Stearns V, Hayes DF (2001) New prognostic factors for breast cancer recurrence. Semin Oncol 28: 53–67 11254867 10.1016/s0093-7754(01)90045-4

[bib24] Keyomarsi K, Tucker SL, Bucholz TA, Callister M, Ding Y, Hortobagyi GN, Bedrosian I, Knickerbocker C, Toyofuku W, Lowe M, Herliczek TW, Bacus SS (2002) Cyclin E and survival in patients with breast cancer. N Engl J Med 347: 1566–1575 12432043 10.1056/NEJMoa021153

[bib25] Le MG, Mathieu M-C, Douc-Rasy S, Le Bihan ML, Abd El All H, Spielmann M, Riou G (1999) c-myc, p53 and bcl-2 apoptosis-related genes in infiltrating breast carcinomas: evidence of a link between bcl-2 protein over-expression and a lower risk of metastasis and death in operable patients. Int J Cancer 84: 562–567 10567899 10.1002/(sici)1097-0215(19991222)84:6<562::aid-ijc4>3.0.co;2-0

[bib26] Leonard RCF (1999) Tumour markers of prognosis. J Clin Oncol 9: 1102–1104 10.1200/JCO.1991.9.7.1102 2045851

[bib27] Loden M, Stighall M, Nielsen NH, Roos G, Emdin SO, Ostlund H, Landberg G (2002) The cyclin D1 and cyclin E high subgroups of breast cancer: separate pathways in tumorigenesis based on pattern of genetic aberrations and inactivation of the Rb node. Oncogene 21: 4680–4690 12096344 10.1038/sj.onc.1205578

[bib28] Lonning PE, Sorlie T, Perou C, Brown P, Botstein D, Borresen-Dale A-L (2001) Microarrays in primary breast cancer – lessons from chemotherapy studies. Endocrine – Related Cancers 8: 259–263 10.1677/erc.0.0080259 11566617

[bib29] Merkel DE, Osborne CK (1989) Prognostic factors in breast cancer. Haem/Oncol Clin N Am 3: 641–651 2558104

[bib30] Mirza AN, Mirza NQ, Vlastos G, Singletary SE (2002) Prognostic factors in node-negative breast cancer: a review of studies with sample sizes more than 200 and follow-up more than five years. Ann Surg 235: 10–26 11753038 10.1097/00000658-200201000-00003 PMC1422391

[bib31] Moliterni A, Menard S, Valagussa P, Biganzoli E, Boracchi P, Balsari A, Casalini P, Tomasic G, Marubini E Pilotti S, Bonadonna G (2003) HER2 overexpression and doxorubicin in adjuvant chemotherapy for resectable breast cancer. J Clin Oncol 21: 458–462 12560435 10.1200/JCO.2003.04.021

[bib32] Niu Y, Fu X, Lu A, Fan Y, Wang Y (2002) Potential markers predicting distant metastases in axillary node-negative breast carcinoma. Int J Cancer 98: 754–760 11920647 10.1002/ijc.10136

[bib33] Pharoah PD, Day NE, Caldas C (1999) Somatic mutations in the p53 gene and prognosis in breast cancer: a meta-analysis. Br J Cancer 80: 1968–1973 10471047 10.1038/sj.bjc.6690628 PMC2363143

[bib34] Porter PL, Malone KE, Heagerty PJ, Alexander GM, Gatti LA, Firpo EJ, Daling JR, Roberts JM (1997) Expression of cell cycle regulators p27^KIP1^ and Cyclin E, alone and in combination, correlate with survival in breast cancer patients. Nat Med 3: 222–225 9018243 10.1038/nm0297-222

[bib35] Robson ME, Chappuis PO, Satagopan J, Wong N, Boyd J, Goffin JR, Hudis C, Roberge D, Norton L, Begin LR, Offit K, Foulkes WD (2004) A combined analysis of outcome following breast cancer: differences in survival based on BRCA1/BRCA2 mutation status and administration of adjuvant treatment. Breast Cancer Res 6: R8–R17 14680495 10.1186/bcr658 PMC314444

[bib36] Ross JS, Fletcher JA (1999) The HER-2/*neu* oncogene: prognostic factor, predictive factor and target for therapy. Sem Cancer Biol 9: 125–138 10.1006/scbi.1998.0083 10202134

[bib37] Schlotter CM, Vogt U, Bosse U, Mersch B, Wassmann K (2003) C-myc, not HER-2/*neu*, can predict recurrence and mortality of patients with node-negative breast cancer. Breast Cancer Res 5: R30–R36 12631396 10.1186/bcr568 PMC154146

[bib38] Schubert CM (2003) Microarray to be used as routine clinical screen. Nat Med 9: 9 10.1038/nm0103-9a 12514705

[bib39] Sjogren S, Inganas M, Lindgren A, Holmberg L, Bergh J (1998) Prognostic and predictive value of c-erbB-2 overexpression in primary breast cancer, alone and in combination with other prognostic markers. J Clin Oncol 16: 462–469 9469329 10.1200/JCO.1998.16.2.462

[bib40] Sotirou C, Powles TJ, Dowsett M, Jazaeri AA, Feldman AL, Assersohn L, Gadizetti C, Libutti SK, Liu ET. (2002) Gene expression profiles derived from fine needle aspiration correlate with response to systemic chemotherapy in breast cancer. Breast Cancer Res 4: R3 12052255 10.1186/bcr433 PMC111028

[bib41] Stewart HJ, Prescott RJ, Forrest APM (2001) Scottish adjuvant tamoxifen trial: a randomised study updated to fifteen years. J Natl Cancer Inst 93: 456–462 11259471 10.1093/jnci/93.6.456

[bib42] van de Vijver M (1993) Molecular changes in breast cancer. Adv Cancer Res 61: 25–56 8346719 10.1016/s0065-230x(08)60954-9

[bib43] van de Vijver MJ, He YD, van't Veer LJ, Dai H, Hart AAM, Voskul DW, Schreiber GJ, Peterse JL, Roberts C, Marton MJ, Parrish M, Atsma D, Witteveen A, Gals A, Delahaye L, van der Velde T, Bartelink H, Rodenhuis S, Rutgers ET, Friend SH, Bernards R (2002) A gene-expression signature as a predictor of survival in breast cancer. N Engl J Med 347: 1999–2009 12490681 10.1056/NEJMoa021967

[bib44] van Slooten H, van de Vijver MJ, van de Velde CJH, van Dierendonck JH (1998) Loss of BCL-2 in invasive breast cancer is associated with high rates of cell death but also with increased proliferative activity. Br J Cancer 77: 789–796 9514059 10.1038/bjc.1998.128 PMC2149956

[bib45] van't Veer LJ, Dai H, van de Vijver MJ, He YD, Hart AAM, Mao M, Peterse HL, van der Kooy K, Marton MJ, Witteveen AT, Schreiber GJ, Kerkhoven RM, Roberts C (2002) Gene expression profiling predicts clinical outcome of breast cancer. Nature 415: 530–536 11823860 10.1038/415530a

[bib46] Venables WN, Ripley BD (2002) Modern Applied Statistics with S-Plus. 4th Edition. Heidelberg: Springer-Verlag

[bib47] Winters ZE, Leek RD, Bradburn MJ, Norbury CJ, Harris AL (2003) Cytoplasmic p21/waf/cip1 expression is correlated with HER-2/neu in breast cancer and is an independent predictor of prognosis. Breast Cancer Res 5: R242–R249 14580260 10.1186/bcr654 PMC314414

[bib48] Yasui Y, Potter JD (1999) The shape of the age-incidence curves of female breast cancer by hormone receptor status. Cancer Causes Control 10: 431–437 10530614 10.1023/a:1008970121595

[bib49] Yoo KY, Tajima K, Miura S, Takeuchi T, Hirose K, Risch H, Dubrow R (1997) Breast cancer risk factors according to combined ER and PgR status: a case–control analysis. Am J Epidemiol 146: 307–314 9270409 10.1093/oxfordjournals.aje.a009271

